# Unprecedented rains decimate surface microbial communities in the hyperarid core of the Atacama Desert

**DOI:** 10.1038/s41598-018-35051-w

**Published:** 2018-11-12

**Authors:** A. Azua-Bustos, A. G. Fairén, C. González-Silva, C. Ascaso, D. Carrizo, M. Á. Fernández-Martínez, M. Fernández-Sampedro, L. García-Descalzo, M. García-Villadangos, M. P. Martin-Redondo, L. Sánchez-García, J. Wierzchos, V. Parro

**Affiliations:** 1 Centro de Astrobiología (CSIC-INTA), 28850 Madrid, Spain; 2 Instituto de Ciencias Biomédicas, Facultad de Ciencias de la Salud, Universidad Autónoma de Chile, Santiago, Chile; 3 Department of Astronomy, Cornell University, Ithaca, 14853 NY USA; 4 Facultad de Ciencias, Universidad de Tarapacá, Arica, Chile; 5 Museo Nacional de Ciencias Naturales (CSIC), 28006 Madrid, Spain

**Keywords:** Phenology, Environmental impact

## Abstract

The hyperarid core of the Atacama Desert, the driest and oldest desert on Earth, has experienced a number of highly unusual rain events over the past three years, resulting in the formation of previously unrecorded hypersaline lagoons, which have lasted several months. We have systematically analyzed the evolution of the lagoons to provide quantitative field constraints of large-scale impacts of the rains on the local microbial communities. Here we show that the sudden and massive input of water in regions that have remained hyperarid for millions of years is harmful for most of the surface soil microbial species, which are exquisitely adapted to survive with meager amounts of liquid water, and quickly perish from osmotic shock when water becomes suddenly abundant. We found that only a handful of bacteria, remarkably a newly identified species of *Halomonas*, remain metabolically active and are still able to reproduce in the lagoons, while no archaea or eukaryotes were identified. Our results show that the already low microbial biodiversity of extreme arid regions greatly diminishes when water is supplied quickly and in great volumes. We conclude placing our findings in the context of the astrobiological exploration of Mars, a hyperarid planet that experienced catastrophic floodings in ancient times.

## Introduction

**Figure 1:**
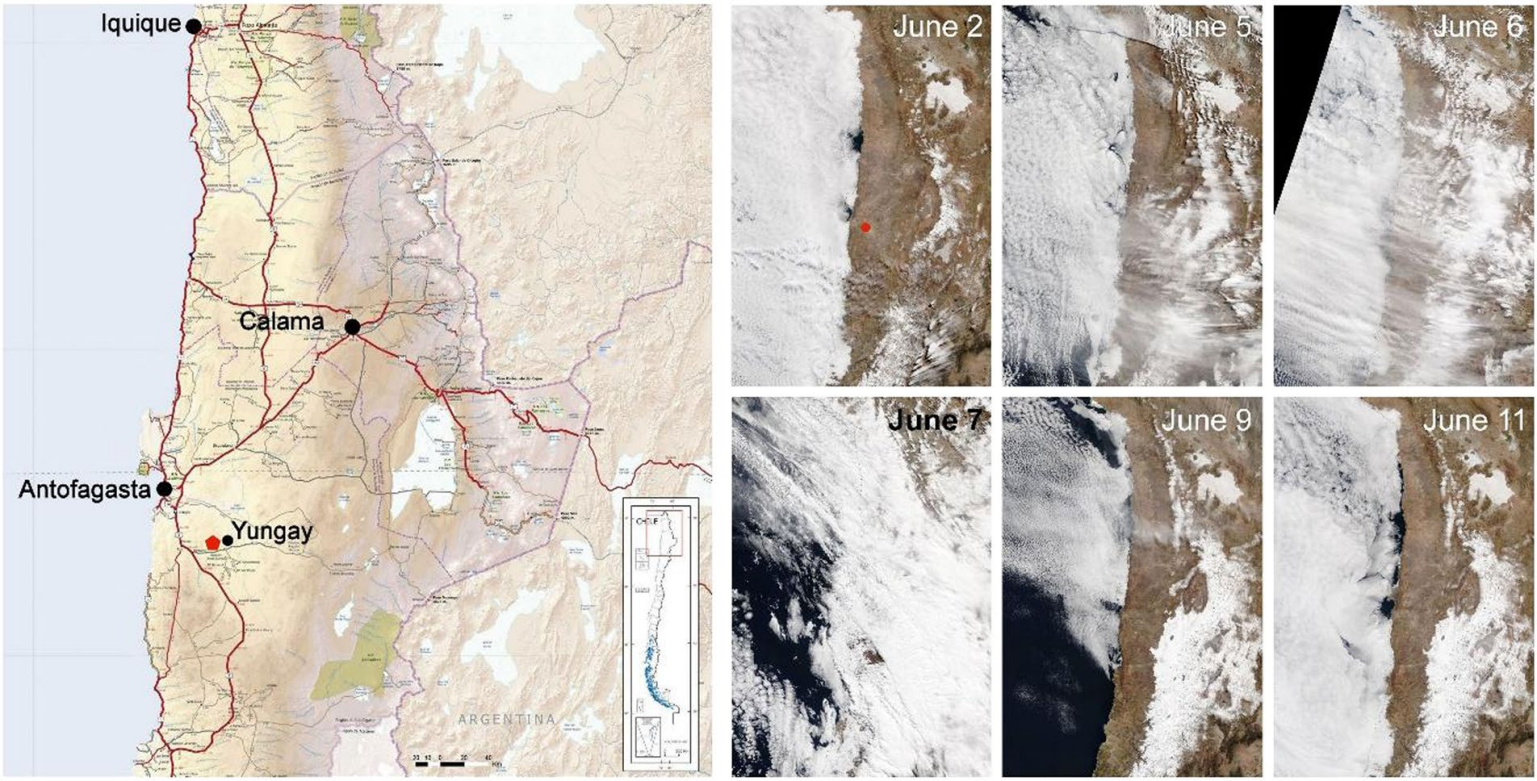
Rains in the Atacama Desert. Left: Map of the Atacama Desert showing the location of the sampled lagoons (red dot). Right: Time lapse satellite images^53^ of the June 2017 rain event. The June 2 panel shows a standard day in the Atacama Desert, with fogs entering the Coastal Range. Note the incoming of an important mass of rain clouds from the Pacific Ocean, with maximum prevalence on June 7, when extensive rains were recorded in the Atacama (June 5 to June 7 panels). The June 9 and June 11 panels show the extensive range of high areas with snow after the June 7 event (compare the June 2 panel with the June 9 panel). For the right panel images, we acknowledge the use of imagery from the NASA Worldview application (https://worldview.earthdata.nasa.gov/) operated by the NASA/Goddard Space Flight Center Earth Science Data and Information System (ESDIS) project^53^.

The Atacama Desert, located in northern Chile, encompasses about 105,000 square kilometers. It is bordered on the east by the Andes Mountains and on the west by the Coastal Range (Fig. 1). The hyperarid core of the Atacama (hereafter “core Atacama”) has remained arid for the past 150 million years, and hyperarid for the past 15 million years^1–5^. Mean annual precipitation in the core Atacama is extremely low, with mean annual values mostly below 4 mm/m^2^ (ref.^6^). Due to this extreme aridity, the Yungay region located in the core Atacama was proposed in 2003 to be a good analog model for Mars studies^1^, and since then, more than 300 reports have detailed the meteorological, geophysical and biological characteristics of the core Atacama^7^. The soils of the core Atacama are highly saline, enriched in nitrates, sulfates and perchlorates^8–10^, and extremely poor in organics^1,5,11,12^. Still, a number of different microbial species from the three domains of life have been reported to inhabit the hyperarid core Atacama; these species are known dry- and radiation-tolerant strains also present elsewhere in the world, and are exquisitely adapted to the extreme desiccating conditions, the high salinity and the high UV radiation^5,7,13–15^ that have been present in the core Atacama for the past 150 million years^3^.

Despite its extreme dryness, part of the Atacama Desert is commonly affected by the “altiplanic winter” between December and March, when moist air comes from the east over the Andes Mountains causing unsettled weather and occasional snow, mostly at the foothills of the Andes at the eastern edge of the core Atacama^16^. Exceptionally, during the past three years, two unique meteorological events have impacted most of the core Atacama: in 2015, two significant rain events were recorded on March 25 and August 9; and in 2017 another one was recorded on June 7 (Table 1). These rain events of 2015 and 2017 originated because extensive mass of clouds entered the Atacama from the Pacific Ocean (from the west) during the last days of autumn (Fig. 1), an unprecedented phenomenon that took place twice in a period of only three years^6^. Including other minor rain events in-between, during the 2015–2017 period mean annual precipitation reached values one order of magnitude higher than the usual for the region, up to 40 mm/m^2^ (Table 1). Climate models suggest that similar rain events could take place once about every century, however there are no records of similar rain events for at least the past 500 years^6,17^.

**Table 1:** Recorded rains (mm/m^2^) in towns nearby Yungay during the last decade.

	Antofagasta	Calama
2008	0	0
2009	2,1	0,9
2010	1,4	1
2011	6,6	10,6
2012	0,6	5,7
2013	0	1,5
2014	1,2	3
2015	38,6	17,1
2016	4,6	6
2017	19,6	3,3
**Total**	**74,7**	**49,1**

**Figure 2:**
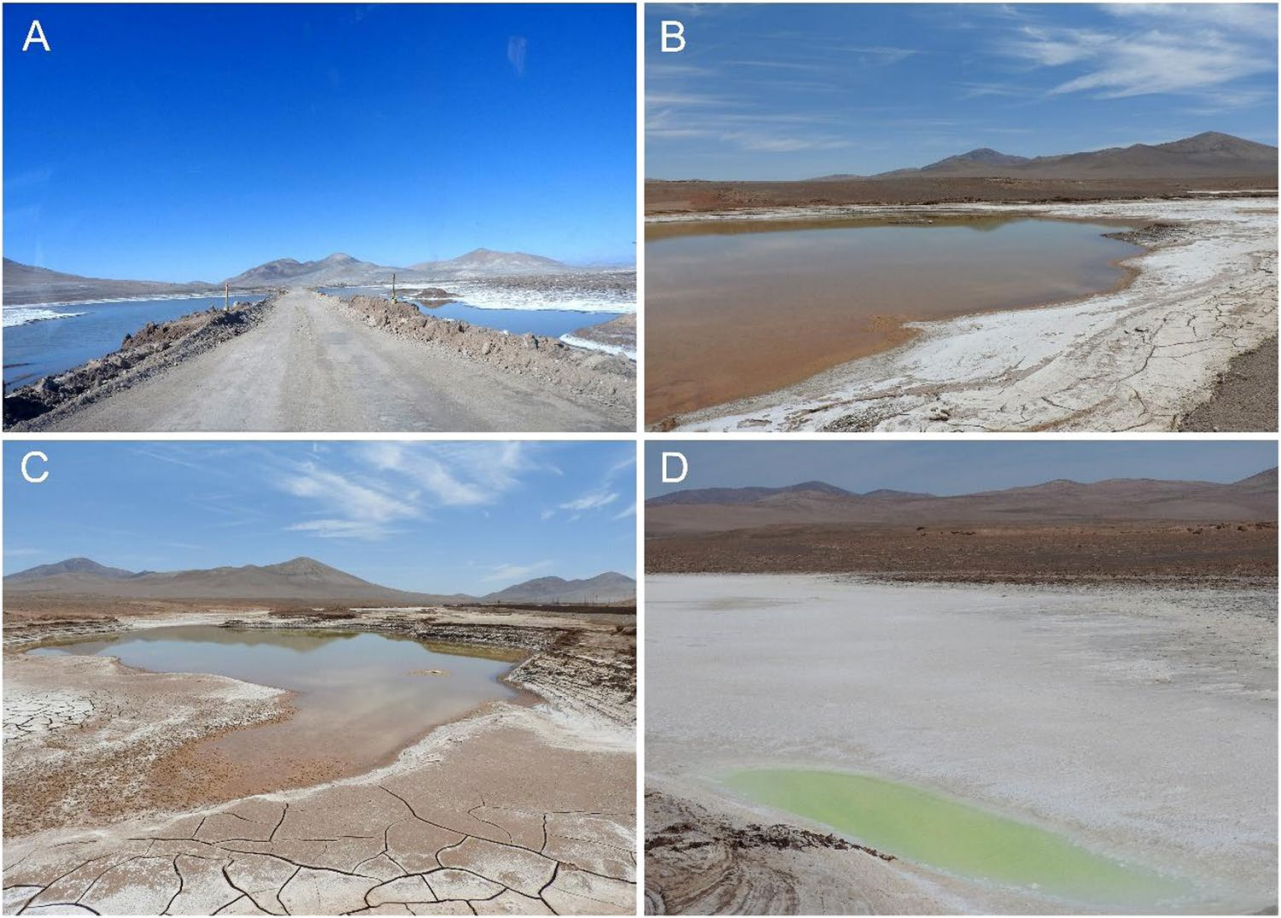
Visual appearance of the lagoons formed after the June 7 2017 rain event in the Yungay region, Atacama Desert. Panel (**A**) shows the lagoons as seen on July 8, 2017. Panels (**B**), (**C**) and (**D**) show the lagoons referred to as *large*, *medium* and *small* in this study, respectively, in pictures taken on November 11, 2017. The small lagoon was characterized by an intense yellow color, probably caused by the important increase in sulfates (see Table 2).

This significant alteration in weather patterns has been attributed to global climate change, with important shifts in rain patterns that have randomly affected different areas of the core Atacama^18^, and with unknown consequences on the composition, physiology and activity of the highly desiccation-adapted microbial species affected. The most noticeable visual effect of these unusual rain events has been the ponding of small lagoons never documented before in the Yungay region (Fig. 2). We sampled three lagoons in Yungay five months after the June 7 2017 rain event, in order to quantitatively assess their volume-dependent characteristics and long-term habitability.

## Results

*In situ* and *ex situ* measurements (ion-exchange chromatography, inductively coupled plasma mass spectrometry and X-Ray Diffraction, see Methods) showed that the physicochemical characteristics of the waters in these lagoons reflected the saline composition and variability of the surrounding soils (Table 2), although the three lagoons have very different Ca and SO_4_ content. Thus, although the salts present in the lagoons were the same than those of the surrounding soils, the water activity of the new watery habitat was much higher. To understand the ecological effects of the water accumulation on the hyperarid soils of Yungay, we systematically examined the lagoons (see Methods) searching for the microbial species that have been previously reported as part of the community of at least 16 endolithic and hypolithic species present in the soils before the rains, which were representative of the three domains of life^5,7,13–15,19^, as follows.

**Table 2:** The studied lagoons in the Atacama Desert: General characteristics (note that mean ocean water salinity is 35 g/Kg); anionic (ion-exchange chromatography) and cationic (inductively coupled plasma (ICP) mass spectrometry) compositions; and species composition (%) of main OTUs (NGS-Based 16S rRNA sequencing, ND: Not Detected). X-Ray Diffraction (XRD) confirmed the presence of sodium chloride (NaCl, halite) and sodium nitrate (NaNO_3_) in both the large- and medium-sized lagoons (Figure S1).

Coordinates	Large	Medium	Small
	24°04′43.1″S, 69°56′35.2″W	24°04′42.5″S, 69°56′28.9″W	24°04′43.0″S, 69°56′02.0″W
Length (m)	60	30	4
Wide (m)	40	20	1
Depth (cm)	15	10	30
pH	6,4 ± 0.02	6,1 ± 0.02	3,8 ± 0.02
EC (mS/cm)	25,06 ± 0.01	28,31 ± 0.01	62,19 ± 0.01
Salinity (g/Kg)	19,8 ± 0.4	21,6 ± 0.4	38,78 ± 0.8
ORP (mV)	−33,5 ± 1	−31 ± 1	−72,7 ± 1
**Anions (ppm)**			
Fluorides	0,09	0,17	0,17
Acetates	0,22	*ND*	0,04
Formiates	*ND*	*ND*	0,02
Chlorides	172,11	1116,01	50,52
Bromides	0,13	0,91	0,41
Nitrates	108,58	654,81	254,10
Sulfates	2,24	31,17	368,89
**Cations (% weight)**			
Na	7,91	7,77	7,71
Mg	0,04	0,04	0,55
K	0,10	0,10	2,13
Ca	0,91	0,92	20,61
**Closest Blast Match**			
Marinimicrobium locisalis	80	43	48
Marinobacter sp.	9	23	52
Halomonas gudaonensis	8	─	─
Acinetobacter sp.	3	34	─

First, we amplified 16S and 18S ribosomal RNA gene sequences, which revealed only the presence of bacteria in the lagoons. No archaea or eukaryotes were found, despite several attempts with archaea and eukaryote specific primers. Massive parallel sequencing of 16S ribosomal RNA gene amplicons showed that more than 60% of the sequences found in the lagoons belonged to only four main OTUs (Operational Taxonomical Units), specifically to the Class Gammaproteobacteria: *Halomonas* (found worldwide^20^), *Marinimicrobium*, *Marinobacter* and *Acinetobacter* (Table 1). A decrease in biodiversity is observed as the salinity of the lagoons increase (Table 1), revealing the higher salinity tolerance of *Marinimicrobium* and *Marinobacter species* compared to that of *Halomonas* and *Acinetobacter* species here reported. Sequences in the OTUs of the remaining 40% were up to 4 orders of magnitude lower, with OTUs with less than 300 sequences discarded as contaminants.

**Figure 3:**
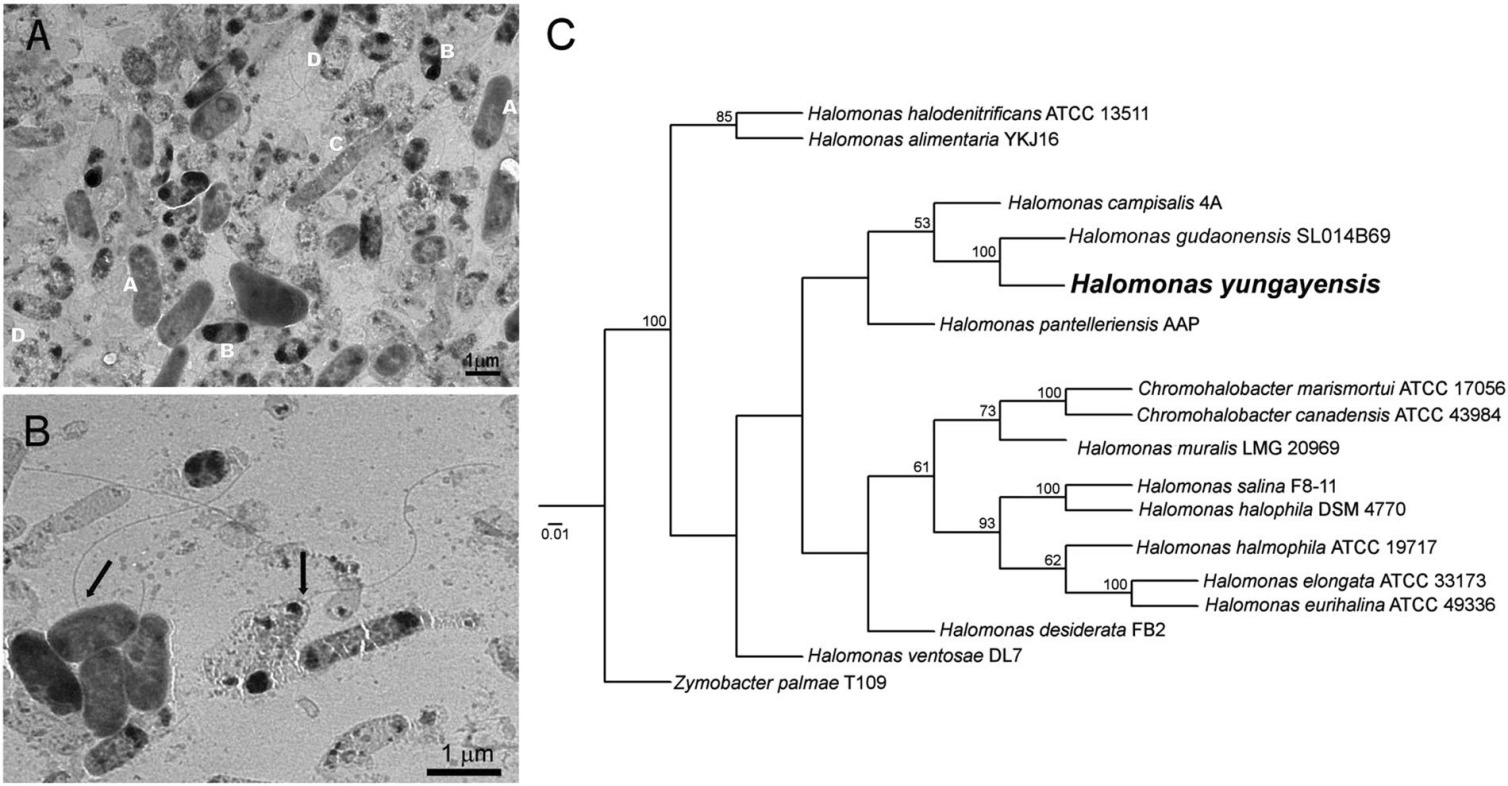
Bacterial species identified in the Atacama lagoons. (**A**) TEM micrograph showing the four distinct detected morphotypes, labeled as A, B, C and D. (**B**) TEM micrograph showing the characteristic single lateral flagella of *Halomonas* (darker cell at left) and single polar flagella of *Marinimicrobium*/*Marinobacter* species (lighter cell at right). (**C**) PHYLIP phylogenetic tree obtained from the aligned 16S rRNA gene of the cultured isolate using BOSQUE. The numbers on the nodes represent bootstrap values with 10000 replicates. Only values over 50% are shown.

Second, we analyzed water samples taken directly from the lagoons with microscopy. Fluorescence microscopy showed the absence of photosynthetic species (Cyanobacteria and microalgae) in the lagoons, a finding further confirmed by bright field microscopy, which did not find microorganisms of the expected sizes and morphologies of cyanobacteria. In agreement with the finding of four main OTUs, our analysis by transmission electron microscopy (TEM) also revealed the presence of at most four different morphotypes (Fig. 3). One of these morphotypes has a single lateral flagella characteristic of *Halomonas gudaonensis*, the closest phylogenetic relative of the *Halomonas* species found in these lagoons. A second morphotype shows the single polar flagella characteristic of *Marinimicrobium* and *Marinobacter* species, while one of the morphotypes also shows two distinctive dark poles, characteristic of *Marinobacter* species.

Third, we cultured samples of the lagoons in different media, detecting growth of a number of isolates only in marine media and only from the samples of the large- and medium-sized lagoons. ERIC PCR fingerprinting^21^ and 16S rRNA sequence phylogenetic analysis of these isolates revealed a single novel bacterial species*, Halomonas yungayensis* sp. nova (Fig. 3). When grown on agar plates, *Halomonas* species typically form white/yellow colonies that turn light brown over time^20^, as it is the case of the new species here reported. We could not grow the bacterial species (*Marinimicrobium*, *Marinobacter*) from the samples of the smallest lagoon in any of the media tested.

And fourth, we conducted biomarker analyses which revealed extremely low concentrations of lipids (Table S1), with no presence of unsaturated n-carboxylic acids, n-alkanols, isoprenoids (like pristane or phytane, typical degradation products of chlorophyll), as well as no sterols of any kind, confirming the absence of eukaryotic cells. Interestingly, we found n-carboxylic acids to be slightly higher in the larger lagoons, consistent with the presence of bacteria. The detection of ramified *n*-carboxylic acid (16-methylheptadecanoic acid iso C18) only in the large- and medium-lagoon is of interest, as unveils not only the presence but also the metabolic activity of bacteria.

Our analyses of the four main OTUs and morphotypes described here show that they correspond with species inhabiting the hyperarid soils of the Atacama Desert before the 2015–2017 rains. *Halomonas* and *Acinetobacter* have been previously found in the soils below and sorrounding these lagoons^19^. *Halomonas* sp. has also been reported in another site located 65 km west, in the Coastal Range of the Atacama, as part of a community of at least 70 other species belonging to the three domains of life^22^. *Halomonas* and *Marinimicrobium* species were also identified as part of a community of about 75 species existing at the El Tatio geyser field at the foothills of the Andes, located 260 km east of Yungay at the eastern edge of the hyperarid core of the Atacama^23^. Besides, *Halomonas* and *Marinobacter* species have been reported as part of a community of about 30 species found in two salt pans located in the northeastern part of the hyperarid core of the desert^24^, helping to confirm that the species reported here are indigenous from the core Atacama. *Halomonas* species are moderately halophilic (explaining their presence in the less saline lagoons), aerobic, gram-negative, rod-shaped and flagellated bacteria^25^. *Marinobacter* species are all halophilic^26^, and essentially require Na^+^ for growing (cannot be replaced by other ions), which explains their increase in relative abundance as the salinity of the lagoons increases (Table 1). Species from the *Marinimicrobium* genus are moderately halotolerant, strictly aerobic, chemoheterotrophic bacteria^27^.

**Figure 4:**
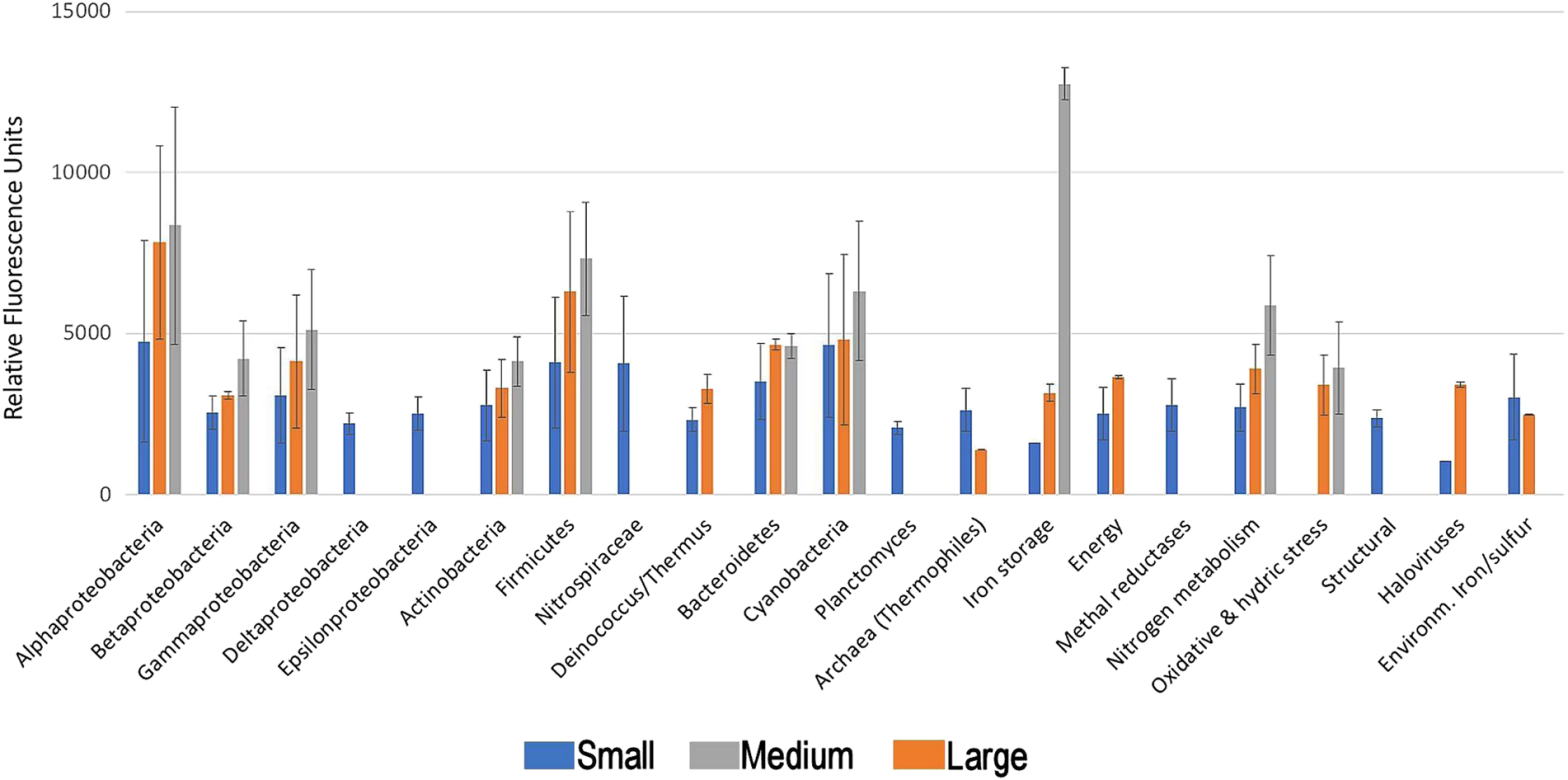
Summary of LDChip results. LDChip showed positive immunodetections associated to different microbial groups. Bars represent the average fluorescence intensity of the different antibodies that showed positive detection from 2 experimental replicates per lagoon and 6 replicate spots per antibody. Error bars correspond to the standard deviation of the average value per group of bacteria or antibody.

Finally, to verify that the soil ecosystems present in the hyperarid Yungay before 2015 have been severely disrupted by the recent rains, we conducted a multiplex antibody microarray LDChip^28^. The immunoassay allowed the detection of a number of biomarkers associated with several microbial phyla (Fig. 4): Alpha, Beta and Gammaproteobacteria, Firmicutes, Actinobacteria (including spores), Bacteroidetes and cyanobacteria of the order Chroococcales. Proteins related to nitrogen fixation, iron metabolism, carbon storage, and even some haloviruses were also detected. The fact that a range of microbial markers were detected using the LDChip, while most of the species from which these biomarkers should come from were not detected by DNA sequence or microscopy, confirmed that these biomarkers are actually remnants of the original microbial community inhabiting the core Atacama before the rain events reported here killed them. This case is well illustrated by the identification of cyanobacteria, which were neither detected by DNA sequencing nor fluorescence, bright field or TEM microscopy, but have been reported as a major component of the original halite communities (Halothece, Chroococcales) of the inspected sites^29^.

## Discussion and Conclusions

### Unprecedented flooding alter the ecological equilibrium of the core Atacama

In the soils of Yungay, and depending on the specific area covered with the inspected lagoons, between 87% and 75% of the previously reported species vanished, with only up to four species of bacteria (two in the most extreme case) able to survive in these new, but transitory, bodies of water. These results of our multiple combined geochemical and microbiological analyses on the newly formed lagoons in the hyperarid core of the Atacama Desert allow us to propose the hypothesis that a massive and sudden input of water in regions that have remained extremely arid for millions of years might cause the disruption of most of the microbial communities that inhabited the surface soils. We suggest that microbial species exquisitely adapted to survive with meager amounts of liquid water^7^ quickly perished from osmotic shock after the flooding. The microbial communities inhabiting below the top 15–20 cm would likely remain unaffected, because they live out of the upper layer analyzed here.

It has not escaped our notice that ecological recovery is possible after the desiccation of the lagoons. The few bacterial groups dominating five months after the rain event could reflect that these groups were well adapted, or were growing the fastest, and outcompeted archaea and eukaryotes may come in later in the recovery sequence. Given that there are archaea or eukaryotes (e.g., *Dunaliella*) well adapted to this sort of mid-range saline environment^30^, it is possible that the initial population after the flood is grown up from the residential soil and salt-dome endolith population. Colonization by new microbial types that would be suitable in the new environment presumably could come later and, over time, the complexity of the flood waters and soils will increase due to incoming species. We are currently sampling the surface and shallow subsurface of soils, both wetted and submerged in water, monitoring the microbial diversity of the saline flood waters over time, and analyzing timepoints since the rain events, to further strengthen our conclusions.

### The core Atacama is a valid analogue for the N cycle and astrobiological studies on Mars

The most useful operational definition of the “core Atacama” is the distribution of nitrate deposits. The 13 My. nitrate deposits^31^ are what lead pioneering researchers to think that there was a hyperarid core in the Atacama Desert, and as such the Yungay site was chosen for investigation because it was near a historic nitrate deposit^32^. In these extremely old, dry and purportedly inactive surfaces nitrates appear to have been moved by fluvial action, yet they are only present in the core Atacama^32^, mostly at the bottom of the valleys and forming roughly equipotential surfaces indicating deposition in standing water^33^. Our geochemical analyses (see Table 2) support previous suggestions^10^ that long periods of dryness build up nitrate deposits uniformly in the soil of the core Atacama, accumulating atmospheric NO_3_^−^; our results further suggest that rare floods, such as those reported here for the first time, wash nitrates down to the valley floors and the water then evaporates before microbial denitrifiers have a chance to deplete the nitrate. In fact, it has been observed^34^ that high nitrate concentrations inhibit denitrification: as the water evaporates, the nitrate gets higher, nitification shuts down because biology cannot consume the nitrate, and the nitrate deposits are formed. Similarly, fixed N has been detected in Mars sediments in the form of nitrates^35^; however, it is still unclear whether a primitive N cycle ever developed on Mars, because the post-depositional behavior of nitrates and the processes capable to recycle oxidized N back into the atmosphere are unknown. Our results in the core Atacama provide the first coherent analog for an incomplete N cycle on Mars: extreme dryness triggering the formation of nitrate deposits, punctuated by extreme flooding concentrating the nitrate in the low-lying areas, and finally evaporation of the flood water before the nitrate can be consumed.

Our results from the Atacama Desert also suggest a possible path for microbiological evolution on early Mars. Mars experienced a complex history of global climate change^36^, including a first period between 4.5 and 3.5 Gyr ago when the planet sustained an active surface hydrosphere, and a subsequent transition to increasingly desiccated conditions, until the Martian surface became the vast dry desert it is today. However, this transition was episodically interrupted by enormous aqueous discharges that flooded regions of the surface on several occasions after 3.5 Gyr ago, and carved the Solar System’s most voluminous channels^37^. In consequence, hypothetical local ecosystems existing in some places on Mars, and adapted to the increasingly dryness of the Mars surface and subsurface after 3.5 Gyr ago^38^, would have been later episodically exposed to even stronger osmotic stresses than those we have reported here for the Atacama microorganisms. As a consequence, the recurrence of liquid water on the surface of Mars after the earliest times might have contributed to decimate local or regional ecosystems, instead of being an opportunity for life to bloom again in the flooded areas, contributing to a heterogeneous distribution of patchy inhabited habitats^39^ during the history of Mars. In addition, the negative results obtained with the life-detecting instruments onboard the 1976 Viking landers^40^ may find the simplest explanation in the fact that, in both the Gas Exchange and Labeled Release experiments, samples were incubated with various watery solutions^41^ with high water activities. Any potential Martian cells would have not been exposed to such elevated values of water activity for at least millions of years, so their sampling and inclusion in the Viking experiments would have caused first their osmotic burst, and then the subsequent destruction of the organic molecules due to the effect of the highly oxidant species characteristic of the Martian regolith^42^.

## Materials and Methods

### Experimental design

Three lagoons located in the Yungay region of the hyperarid core of the Atacama Desert were sampled on November 11, 2017 (coordinates detailed in Table 2). Water samples were taken with sterile gloves and sterile 50 ml falcon tubes, and kept at room temperature for further processing. For all experiments, at least triplicates were analyzed, and in most cases up to 10 samples per lagoon were analyzed.

### Ion Chromatography

Water samples were loaded into a Metrohm 861 Advanced compact ion chromatographer IC (Metrohm AG, Herisau, Switzerland) by an automatic loader, undiluted or at different dilution values, depending on the expected ion concentration. Samples were diluted in IC-grade water (Sigma Aldrich). For all anions, the column Metrosep A supp 7–250 was used with 3.6 mM sodium carbonate (NaCO_3_) as eluent. Each sample was measured three times, and each measurement at a different dilution, to take the values that best fitted the calibration curve. The measurement error of the equipment for replicate samples was less than 1%. The instrument was calibrated with a multi-anionic solution with 6-point concentrations curve for each anion and detection limits at few ppb level for all of them.

### Inductively Coupled Plasma Mass Spectrometry (ICP-MS)

Quantitative analysis of Mn, Cu, Co, Cd, Ba, Na, Mg, K, Ca, Fe, Ni, Zn, and As were performed using a PerkinElmer NexION 2000 ICP-MS (PerkinElmer Inc.) using the conditions shown in Table S2. A semiquantitative analysis using 47 elements as external standards, detailed in Table S3, was previously performed to determine concentration levels prior to quantification. The quadrupole cell allows the adjustment of ion transmission of any isotope without affecting other masses. This capability was applied to Na and K, since both elements have low ionization potentials, high isotopic abundances and are present at high levels in the water samples analyzed. The electronic dilutions conditions are detailed in Table S4. The instrument was tuned to maximum sensitivity and the lowest background, oxides and double charge ion prior to the analysis using a solution containing 1 μg/L Be, Ce, Fe, In, Li, Mg, Pb and U. Samples were taken up by an ASX-500 CETAC Autosampler and on-line addition of internal standard. Helium (99.9999%) was used as collision gas (KED mode) to remove possible polyatomic interferences. Gas conditions are detailed in Table S5. External calibration involved eight solutions from 1 to 5000 ppb, prepared diluting 1000 ppm monoelemental standard solution (SCP Science) in volumetric flask in 1% nitric solution prepared with high purity MilliQ system water and Suprapur grade nitric acid (Merck). All external calibration equations achieved a minimum correlation coefficient of 0.999. Solutions were prepared by diluting 0.02 ml of each lagoon sample to 10 ml with maximum accuracy. A Quality control standard containing 100 ppb of all elements was measured at the end, to control the standard recovery and equipment deviation.

### X-Ray Diffraction

In order to characterize the mineralogical composition of the lagoons, water samples from all lagoons were inspected by X-Ray Diffraction, using a Bruker D8 Eco Advance with Cu Kα radiation (λ = 1.542 Å) and Lynxeye XE-T linear detector. The X-ray generator was set to an acceleration voltage of 40 kV and a filament emission of 25 mA. Samples were scanned between 5° (2θ) and 70° (2θ) using a step size of 0.1° (2θ) and a count time of 1 s, using the Bragg–Brentano geometry. The analysis of the XRD diffraction spectra (diffractograms) of powdered crystals obtained from the evaporation of 5 ml of the large and medium sized lagoons was performed with the DIFFRAC.EVA software (Bruker AXS).

### GC-MS Analysis

The three lipidic fractions (non-polar, acid and polar fraction) present in water samples of the lagoons were analyzed by gas chromatography mass spectrometry using a 6850 GC system coupled to a 5975 VL MSD with a triple axis detector (Agilent Technologies), operating with electron ionization at 70 eV and scanning from *m/z* 50 to 650. The analytes were injected (1 μl) and separated on a HP-5MS column (30 m × 0.25 mm i.d. ×0.25 um film thickness) using He as a carrier gas at 1.1 ml min^−1^. For the non-polar fraction, the oven temperature was programmed to increase from 50 C to 130 °C at a rate of 20 °C/min, then to 300 °C at 6 °C/min (held 20 min). For the acid fraction the oven temperature was programmed from 70 °C to 130 °C, at 20 °C/min; and to 300 °C at 10 °C/min (held 10 min). For the polar fraction, the oven temperature program was the same as for the acid fraction, but the oven was held for 15 min at 300 °C. Injector temperature was 290 °C, transfer line 300 °C and MS source at 240 °C. Compound identification was based on the comparison of mass spectra and/or reference compounds, and compounds were quantified using external calibration curves. External standards of *n*-alkanes (C_10_ to C_40_), FAMEs (C_8_ to C_24_), alcohols (C_10_, C_14_, C_18_, C_20_) and branched isoprenoids (2,6,10-trimethyl-docosane, crocetane, pristane, phytane, squalane and squalene) were injected to obtain calibration curves. Recoveries of the internal standards averaged 75 ± 15%.

### Illumina NGS-Based 16S rRNA Sequencing

DNA extraction: Samples from the lagoons were centrifuged for 5 min at 13000 rpm, and the supernatants carefully discarded in order to collect the resultant pellets. DNA was extracted from these pellets using the DNeasy PowerSoil Kit according the manufacturer instructions, except that at the cell lysis step, one pulse of 2 minutes was used in a FastPrep-24 5 G homogenizer (MP Biomedicals), to better preserve DNA integrity.

Purified DNAs were then amplified in a first PCR of 30 cycles with Q5® Hot Start High-Fidelity DNA Polymerase (New England Biolabs) in the presence of 100 nM primers for 16S amplification (5′-ACACTGACGACATGGTTCTACACCTACGGGNGGCWGCAG-3′ and 5′-TACGGTAGCAGAGACTTGGTCTGACTACHVGGGTATCTAATCC-3′, these primers amplify the V3-V4 region of 16S). After the first PCR, a second PCR of 15 cycles was performed with Q5® Hot Start High-Fidelity DNA Polymerase (New England Biolabs) in the presence of 400 nM of primers 5′-AATGATACGGCGACCACCGAGATCTACACTGACGACATGGTTCTACA-3′ and 5′-CAAGCAGAAGACGGCATACGAGAT-[10 nucleotides barcode]-TACGGTAGCAGAGACTTGGTCT-3′) of the Access Array Barcode Library for Illumina Sequencers (Fluidigm).

The obtained amplicons were validated and quantified by a Bioanalyzer, and an equimolecular pool was purified using AMPure beads and titrated by quantitative PCR using the “Kapa-SYBR FAST qPCR kit for Light Cycler 480” and a reference standard for quantification. The pool of amplicons was denatured prior to be seeded on a flowcell at a density of 10pM, where clusters were formed and sequenced using a “MiSeq Reagent Nano Kit v2”, in a 2 × 250 pair-end sequencing run on a MiSeq sequencer”.

The obtained raw sequences were processed in MOTHUR software v.1.40.0 (ref.^43^), using a custom script based upon MiSeq SOP (ref.^44^). Sequence reads were clustered into OTUs (Operational Taxonomic Units) at the 97% similarity level. Datasets were rarefied independently by random selection to even sequencing depth, corresponding to the lesser number of sequences found in the samples (60673 reads). Taxonomic affinities for the reads were assigned by comparison of OTUs representative sequences against RDP (RDP reference files v.16; release 11 (ref.^45^)) and against nr/nt (NBCI) databases. OTU’s affinities reported as ‘cyanobacteria/chloroplast’ were further assigned a taxonomic identity by comparing them against EMBL, Greengenes and SILVA databases for identification. Sequences assigned to mitochondria or chloroplasts were removed from further analyses as contaminants, as fluorescence microscopy did not detect chlorophyll autofluorescence in any of the sampled lagoons.

### Cultivation and identification of isolates

One ml of each lagoon was aerobically incubated at room temperature (~25 °C) in Petri dishes containing agar and three different growing media: Luria Broth (Sigma), Nutrient agar (Pronadisa) and Marine Media (Conda). Growth was followed during 2 weeks.

### DNA extraction from isolates

DNA was extracted as detailed for Illumina NGS-Based 16S rRNA Sequencing.

### ERIC-PCR fingerprinting

This technique was used in order to detect the number of unique isolates from all the colonies that grew in marine media. ERIC-PCR uses specific primers that amplify ERIC (Entero-Bacterial Repetitive Intergenic Consensus) sequences, giving as a result a number of bands of different sizes that is unique for each bacterial species. ERIC PCR was first used to characterize enteric species^46^, but was subsequently found to be useful for other types of bacteria too^21^. DNA was amplified using the GoTaq Green Master Mix (Promega), using the primers ERIC2 5′AAGTAAGTGACTGGGGTGAGCG3′ and ERIC1R 5′-ATGTAAGCTCCTGGGGATTCAC-3′. PCR conditions used were: 95 °C for 2 min, 92 °C for 30 s, and 35 cycles of (92 °C for 30 s, 48 °C for 80 s, and 65 °C for 108 s), followed by 68 °C for 8 min. The resultant reaction was visualized in a 2% agarose gel at 50 V. Based on the number and molecular weight of bands of the isolates observed in 2% TAE agarose gels, only a single isolate was detected.

### 16S rRNA amplification and sequencing of distinct isolates

16S rRNA of isolates was amplified using the GoTaq Green Master Mix (Promega) and the primers Bac8f AGAGTTTGATCATGGCTCAG and UN1541 AAGGAGGTGATCCAACC. PCR conditions used were: 95 °C for 5 min, and 25 cycles of (95 °C for 40 s, 55 °C for 2 min, 72 °C for 1 min) followed by 72 °C for 7 min. The resultant reaction was visualized in a 2% agarose TAE gel at 50 V.

The automated sequencing of the resulting PCR products was conducted by Macrogen DNA Sequencing Inc. (Seoul, Korea).

### Isolates Phylogeny

Closest species of the isolate obtained was determined by analyzing the 16S rRNA gene sequences obtained using the Megablast option for highly similar sequences of the BLASTN algorithm against the National Centre for Biotechnology Information nonredundant database (www.ncbi.nlm.nih.gov).

Phylogenetic analysis of 16S rRNA gene sequences were aligned by multiple sequence comparison by log-expectation (MUSCLE)^47^, analyzed with jModelTest^48^ and then by Phylip NJ^49^, all tools of the BOSQUE phylogenetic analysis software^50^, as similarly performed in previous works^5,21^.

### Transmission Electron Microscopy

As the objective was to examine the aspect of the species detected by 16S rRNA, we analyzed samples from the small and large lagoons, because we knew that all four species found were present in the large lagoon. Therefore, for convenience, we examined the large and small lagoon samples only, knowing that the medium sized lagoon samples were an intermediate situation.

For negative staining, a solution of sodium phosphotungstate (Sigma, ref, P-6395) was employed. 200 mesh copper grids covered with formvar and reinforced with carbon were used. Cultures were centrifuged and washed twice with ammonium acetate, pH 7, 0,1 M. Pellets were resuspended in ammonium acetate solution, until the concentration of cells was adequate to produce no excess material at the time of observation. Sodium phosphotungstate salt was prepared at 1% (dry wt/vol), and its pH adjusted to 7 with NaOH. A volume of cells were mixed with the sodium phosphotungstate solution. Small drops of this mixture were placed on Parafilm and the grids were floated on it for 5 minutes. Then, the remaining material in the grids was dried, and the grids were placed for five additional minutes on a drop of distilled water. Finally, grids were removed, allowed to dry and observed by Transmission Electron Microscopy (TEM) (JEOL, JEM-2100 instrument with a LaB6 filament, operating at 200 kV acceleration potential).

The characterization of different bacterial morphotypes was achieved by the determination of distinct and different micromorphologies, as detailed in ref.^51^. Briefly, the four detected morphotypes were found by examining all TEM micrographs obtained and inspecting for unique morphologies (i.e., size, shape, presence or absence of flagella, number of flagella, position of insertion of the flagella, presence/absence of electron-dense bodies and its location). In order to confirm these findings, we then compared these morphotypes with the morphologies reported for the species identified by 16 rRNA, finding a coincidence for all four cases.

It is unlikely that cyanobacteria could have not been observed due to 16S primer region mismatches and in addition preferentially lost during the microscopy sample preparation procedure, because no cyanobacteria were observed after a detailed inspection by bright field microscopy of water samples taken directly from the analyzed lagoons. They were not observed by TEM and they did not appear in the molecular data, so we are confident on this negative result.

### Lipid Extraction, Fractionation and Analysis

Water samples were filtered through a GFF pre-cleaned filter, then extracted with a mixture of dichloromethane/methanol (DCM/MeOH, 3:1, v/v) with an ultrasound apparatus (3 × 30 min cycles at room temperature). Internal standards (tetracosane-D_50_, myristic acid D_27_, 2-hexadecanol) were added prior to extraction. Total lipids extracts were concentrated using rotary evaporation to 2 ml. After this step, activated Cu was added and left overnight for elemental sulfur removal. The extracted sample was separated in three fractions using a Bond-elute column chromatography (Bond phase NH_2_, 500 mg, 40 μm particle size). The neutral lipid fraction was obtained by eluting with 15 ml DCM/2-propanol (2:1, v/v), the acid fraction with 15 ml of acetic acid (2%) in diethyl ether, and the phospholipid fraction with 15 ml of methanol. Further separation of the neutral lipid fraction was completed using 0.5 g of alumina in a Pasteur pipe. The non-polar fraction was obtained by eluting 4.5 ml of hexane/DCM (9:1, v/v), and the polar fraction with 3 ml of DCM/methanol (1:1, v/v). The acid fraction was derivatized with BF_3_ in methanol, and the polar fraction with N,O-bis (trimethylsilyl) trifuoroacetamide (BSTFA).

### Fluorescence Sandwich Microarray Immunoassay with a Life Detector Chip (LDChip)

A LDChip containing 200 antibodies to crude lysates of bacterial and archaeal strains, as well as to key proteins and peptides from different universal metabolisms as nitrogen and carbon fixation, iron metabolism (oxidation, reduction, storage), sulfur oxidation, or methanogenesis, was used to detect and profile microbial markers in the three studied lagoons. The water samples were processed and analyzed by multiplex sandwich microarray immunoassay with LDChip as described previously^27^. The anti-cyanobacterial antibodies are specific^52^. A confirmation of this result is that the amplification of 16S rRNA with cyanobacteria-specific primer and the microscopy analysis of the samples in search of cyanobacteria turned both negative.

## Electronic supplementary material

Supplementary Information

## Data Availability

The authors declare that all the data supporting the findings of this study are available within the article (and its Supplementary Information file), or available from the corresponding authors on reasonable request.
