# Author Correction: Unprecedented rains decimate surface microbial communities in the hyperarid core of the Atacama Desert

**DOI:** 10.1038/s41598-019-40319-w

**Published:** 2019-05-21

**Authors:** A. Azua-Bustos, A. G. Fairén, C. González-Silva, C. Ascaso, D. Carrizo, M. Á. Fernández-Martínez, M. Fernández-Sampedro, L. García-Descalzo, M. García-Villadangos, M. P. Martin-Redondo, L. Sánchez-García, J. Wierzchos, V. Parro

**Affiliations:** 1 Centro de Astrobiología (CSIC-INTA), 28850 Madrid, Spain; 2 Instituto de Ciencias Biomédicas, Facultad de Ciencias de la Salud, Universidad Autónoma de Chile, Santiago, Chile; 3 Department of Astronomy, Cornell University, Ithaca, 14853 NY USA; 4 Facultad de Ciencias, Universidad de Tarapacá, Arica, Chile; 5 Museo Nacional de Ciencias Naturales (CSIC), 28006 Madrid, Spain

Correction to: *Scientific Reports* 10.1038/s41598-018-35051-w, published online 12 November 2018

This Article contains typographical errors in the Results section where,

“Massive parallel sequencing of 16S ribosomal RNA gene amplicons showed that more than 60% of the sequences found in the lagoons belonged to only four main OTUs (Operational Taxonomical Units), specifically to the Class Gammaproteobacteria: Halomonas (found worldwide^20^), Marinimicrobium, Marinobacter and Acinetobacter (Table 1). A decrease in biodiversity is observed as the salinity of the lagoons increase (Table 1), revealing the higher salinity tolerance of Marinimicrobium and Marinobacter species compared to that of Halomonas and Acinetobacter species here reported.”

should read:

“Massive parallel sequencing of 16S ribosomal RNA gene amplicons showed that more than 60% of the sequences found in the lagoons belonged to only four main OTUs (Operational Taxonomical Units), specifically to the Class Gammaproteobacteria: Halomonas (found worldwide^20^), Marinimicrobium, Marinobacter and Acinetobacter (Table 2). A decrease in biodiversity is observed as the salinity of the lagoons increase (Table 2), revealing the higher salinity tolerance of Marinimicrobium and Marinobacter species compared to that of Halomonas and Acinetobacter species here reported.”

