# Supplementary material for: Unprecedented rains decimate surface microbial communities in the hyperarid core of the Atacama Desert
Source: Sci Rep. 2018 Nov 12;8:16706. doi: 10.1038/s41598-018-35051-w (PMC6232106; doi:10.1038/s41598-018-35051-w)
Supplement: Supplementary file 1 — Supplementary Information [file 41598_2018_35051_MOESM1_ESM.docx]

Supplementary Materials for

Unprecedented rains are damaging for surface microbial communities in the hyperarid core of the Atacama Desert

A. Azua-Bustos^1,2*^, A. G. Fairén^1,3*^, C. González Silva^4^, C. Ascaso^5^, D. Carrizo^1^, M.Á. Fernández-Martínez^1^, M. Fernández-Sampedro^1^, L. García-Descalzo^1^, M. García-Villadangos^1^, M.P. Martin-Redondo^1^, L. Sánchez-García^1^, J. Wierzchos^5^, and V. Parro^1^

**Affiliations:**

^1^ Centro de Astrobiología (CSIC-INTA), 28850 Madrid, Spain.

^2^ Instituto de Ciencias Biomédicas, Facultad de Ciencias de la Salud, Universidad Autónoma de Chile, Santiago, Chile.

^3^ Department of Astronomy, Cornell University, Ithaca 14853 NY, USA.

^4^ Centro de Investigación del Medio Ambiente (CENIMA), Universidad Arturo Prat, Iquique, Chile.

^5^ Museo Nacional de Ciencias Naturales (CSIC), 28006 Madrid, Spain.


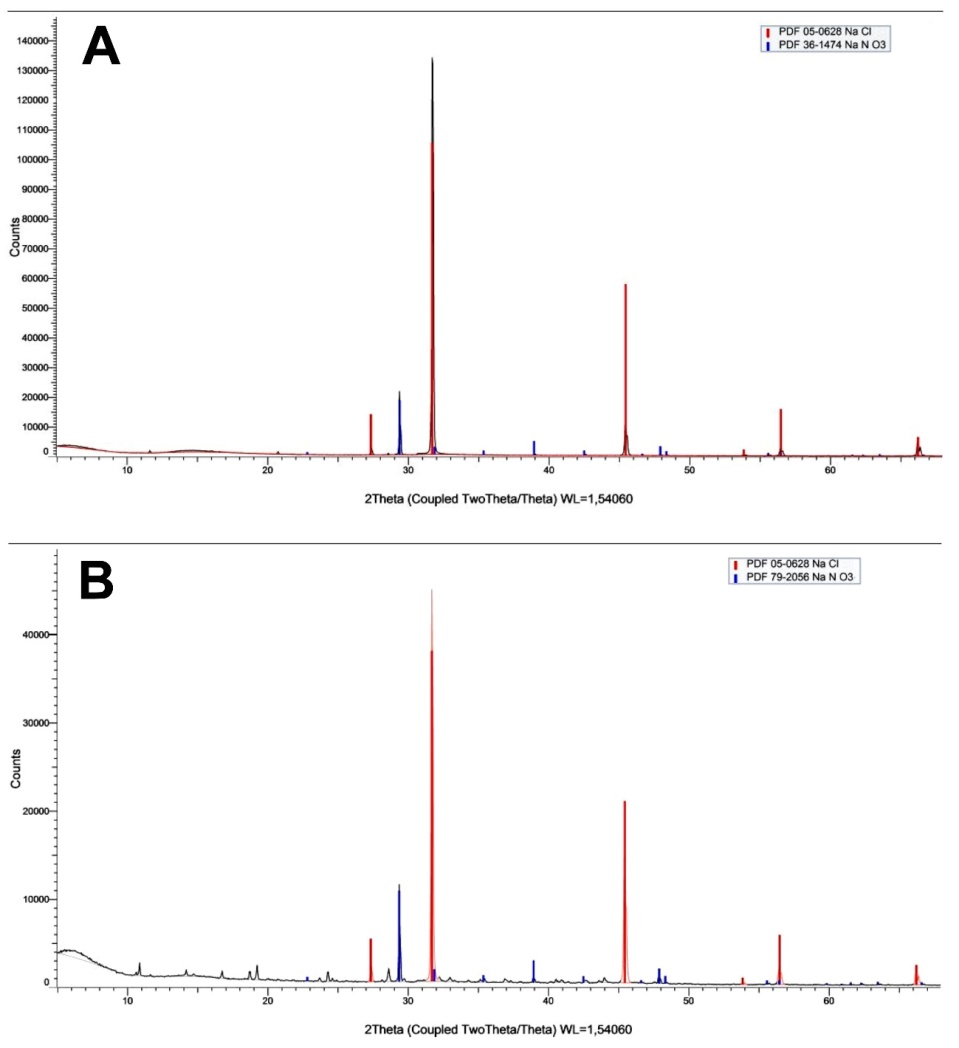


**Fig. S1.** XRD graphs of the sampled lagoons. (A) Large lagoon. (B) Medium sized lagoon. XRD could not be performed in samples of the small lagoon because its extreme salinity maintained these samples liquefied despite several crystallization attempts, suggesting the presence of perchlorates^54^.

**Table S1.** Lipid biomarkers identified in the lagoons. (nd: not detected).

|  |  |  |  |  |  |
| --- | --- | --- | --- | --- | --- |
|  | **Lipid Biomarker** | **Small** | **Medium** | **Large** |  |
|  | *n-alkanes* |  |  |  |  |
|  | Heptadecane (C19) | nd | 0,001 | nd |  |
|  | Eicosane (C20) | 0,002 | 0,001 | 0,003 |  |
|  | Heneicosane (C21) | 0,001 | 0,005 | 0,004 |  |
|  | Docosane (C22) | 0,002 | 0,002 | 0,002 |  |
|  | Tricosane (C23) | 0,005 | 0,012 | 0,010 |  |
|  | Tetracosane (C24) | 0,002 | 0,002 | 0,007 |  |
|  | Pentacosane (C25) | 0,012 | 0,013 | 0,012 |  |
|  | Hexacosane (C26) | 0,005 | 0,014 | 0,012 |  |
|  | Heptacosane (C27) | 0,026 | 0,029 | 0,034 |  |
|  | Octacosane (C28) | 0,011 | 0,013 | 0,015 |  |
|  | Nonacosane (C29) | 0,035 | 0,035 | 0,043 |  |
|  | Triacontane (C30) | 0,017 | 0,014 | 0,018 |  |
|  | Hentriacontane (C31) | 0,024 | 0,038 | 0,022 |  |
|  |  |  |  |  |  |
|  | Pristane (2,4,10,14-tetramethylpentadecane) | nd | nd | nd |  |
|  | Phytane (2,4,10,14-tetramethylhexadecane) | nd | nd | nd |  |
|  |  |  |  |  |  |
|  | *n-carboxylic acids* |  |  |  |  |
|  | Hexadecanoic acid (C16:0) | 0,06 | 0,16 | 0,12 |  |
|  | 16-methylheptadecanoic acid (iso C18) | nd | 0,05 | 0,03 |  |
|  | Octadecanoic acid (C18:0) | 0,09 | 0,18 | 0,15 |  |
|  | 14-methylhexadecanoic acid (anteiso C17) | nd | nd | nd |  |
|  | Heptadecanoic acid (C17:0) | nd | nd | nd |  |
|  | 15-methylheptadecanoic acid (anteiso C18) | nd | nd | nd |  |
|  | 15-methylhexadecanoic acid (iso C17) | nd | nd | nd |  |
|  |  |  |  |  |  |
|  | *n-alkanols* |  |  |  |  |
|  | Dodecanol (C22) | nd | nd | nd |  |
|  | Tridecanol (C23) | nd | nd | nd |  |
|  | Tetradecanol (C24) | nd | nd | nd |  |
|  | Pentadecanol (C25) | nd | nd | nd |  |
|  | Hexadecanol (C26) | nd | nd | nd |  |
|  | Heptadecanol (C27) | nd | nd | nd |  |
|  | Octadecanol (C28) | nd | nd | nd |  |
|  |  |  |  |  |  |
|  | *Sterols* |  |  |  |  |
|  | Cholesterol | nd | nd | nd |  |
|  | Stigmastanol | nd | nd | nd |  |
|  | β-Sitosterol | nd | nd | nd |  |
|  |  |  |  |  |  |

**Table S2.** Inductively coupled plasma mass spectrometry (ICP-MS) conditions.

|  |  |  |
| --- | --- | --- |
|  | **Component/Parameter** | **Type/value** |
|  | Nebulizer | MEINHARD® pluss Glass Type C |
|  | Spray Chamber(Peltier-cooled) | Glass Cyclonic At 5°C |
|  | Sampler and skimmer cones | Nickel |
|  | Sample Uptake Rate | 350μL/min |
|  | PF Power | 1600 W |
|  | Injector | 2.0 mm Id quartz |
|  | Sweeps | 20 |
|  | Dwell Time | 25 |
|  | Replicates | 3 |
|  | Mixing Tee | On-line addition of internal standard |
|  |  |  |

**Table S3.** Standards used in ICP-MS.

| ***Element*** | **External Standard1 (ppb)** | **QC.Std1 (ppb)** |
| --- | --- | --- |
| *Ag* | 50 | 100 |
| *Al* | 49 | 98 |
| *As* | 490 | 980 |
| *B* | 490 | 980 |
| *Ba* | 48.5 | 97 |
| *Be* | 510 | 1020 |
| *Bi* | 49 | 98 |
| *Ca* | 4945 | 9890 |
| *Cd* | 49.5 | 99 |
| *Co* | 49.5 | 99 |
| *Cr* | 49.5 | 99 |
| *Cu* | 49.5 | 99 |
| *Fe* | 500 | 1000 |
| *Ga* | 49.5 | 99 |
| *K* | 49 | 98 |
| *Li* | 49.5 | 99 |
| *Mg* | 49 | 98 |
| *Mn* | 49.5 | 99 |
| *Mo* | 49.5 | 99 |
| *Na* | 48.5 | 97 |
| *Ni* | 49.5 | 99 |
| *Pb* | 49 | 98 |
| *Rb* | 49.5 | 99 |
| *Se* | 505 | 1010 |
| *Sr* | 500 | 1000 |
| *Te* | 49.5 | 99 |
| *Tl* | 49.5 | 99 |
| *U* | 49 | 98 |
| *V* | 49 | 98 |
| *Zn* | 495 | 990 |
| *Ce* | 50 | 100 |
| *Dy* | 50 | 100 |
| *Er* | 50 | 100 |
| *Eu* | 50 | 100 |
| *Gd* | 50 | 100 |
| *Ho* | 50 | 100 |
| *La* | 50 | 100 |
| *Lu* | 50 | 100 |
| *Nd* | 50 | 100 |
| *Pr* | 50 | 100 |
| *Sm* | 50 | 100 |
| *Sc* | 50 | 100 |
| *Tb* | 50 | 100 |
| *Th* | 50 | 100 |
| *Tm* | 50 | 100 |
| *Y* | 50 | 100 |
| *Yb* | 50 | 100 |

**Table S4.** ICP-MS electronic dilutions conditions.

|  | **Element** | **Mass** | **Abundance** | **RPa value** |
| --- | --- | --- | --- | --- |
|  | Na | 23 | 100% | 0.018 |
|  | K | 39 | 93% | 0.015 |
|  |  |  |  |  |

**Table S5.** ICP-MS gas conditions.

|  | **Element** | **Isotope analyzed** | **He flow (KED mode)(l/min)** |  |
| --- | --- | --- | --- | --- |
|  | Al | 27 | 1 |  |
|  | Mn | 55 | 1 |  |
|  | Cu | 65 | 1 |  |
|  | Co | 59 | 1 |  |
|  | Cd | 111 | 1 |  |
|  | Ba | 137 | 1 |  |
|  | Pb | 208 | 1 |  |
|  | U | 238 | 1 |  |
|  | Na | 23 | 4 |  |
|  | Mg | 24 | 4 |  |
|  | K | 39 | 4 |  |
|  | Ca | 44 | 4 |  |
|  | Cr | 52 | 4 |  |
|  | Fe | 56 | 4 |  |
|  | Ni | 60 | 4 |  |
|  | Zn | 66 | 4 |  |
|  | As | 75 | 4 |  |
|  | P | 31 | 4 |  |
